# Quantitative 3-Dimensional Characterization of Aortic and Carotid Artery Tortuosity in Mucopolysaccharidosis Type IVa: An Exploratory Comparative Imaging Study

**DOI:** 10.3390/diagnostics16213627

**Published:** 2026-11-03

**Authors:** André Lollert, Seyfullah Gökce, Christian Hoffmann, Fabio Souschek, Heidi Rossmann, Friederike Häuser, Lisa Maiurano, Gundula Staatz, Julia B. Hennermann

**Affiliations:** 1 Section of Pediatric Radiology, Department of Diagnostic and Interventional Radiology, University Medical Center of the Johannes Gutenberg University, 55131 Mainz, Germany; 2 Center of Pediatric and Adolescent Medicine, Villa Metabolica, University Medical Center of the Johannes Gutenberg University, 55131 Mainz, Germany; 3 Pediatric Practice Gökce, 65185 Wiesbaden, Germany; 4 Institute of Clinical Chemistry and Laboratory Medicine, University Medical Center of the Johannes Gutenberg University, 55131 Mainz, Germany

**Keywords:** mucopolysaccharidosis type IVa, Morquio disease, tortuosity, MR angiography

## Abstract

**Background/Objectives**: Vascular involvement has been described in mucopolysaccharidosis type IVa (MPS IVa), but the morphology of the aorta and carotid arteries has not been comprehensively characterized. This study aimed to quantitatively assess aortic and carotid artery tortuosity and kinking using contrast-enhanced three-dimensional (3D) magnetic resonance (MR) angiography. **Methods**: In this retrospective exploratory comparative imaging study, six patients with confirmed MPS IVa underwent clinically indicated 3D MR angiography of the aorta and supraaortic vessels; two were excluded due to motion artifacts, leaving four patients for analysis. Two radiologists independently assessed vascular kinking and tortuosity. For comparison, 12 age- and sex-matched polytrauma patients without documented vascular abnormalities were retrospectively evaluated using computed tomography angiography. **Results**: All four patients with MPS IVa showed multiple kinking sites of the descending aorta and increased tortuosity of the common and internal carotid arteries. Aortic tortuosity indices ranged from 2.01 to 2.68. Carotid tortuosity indices were also increased, particularly in the internal carotid arteries. Compared with controls, all assessed tortuosity indices were consistently higher in patients with MPS IVa (exploratory *p* = 0.001). Despite multiple aortic kinking sites, no marked anatomical luminal narrowing was observed. **Conclusions**: This exploratory study provides quantitative evidence of differences in aortic and carotid geometry between a small, highly selected MPS IVa cohort and external imaging controls. Larger studies using harmonized imaging protocols and longitudinal follow-up are needed to assess prevalence, disease-specific causation and clinical significance of these findings.

## 1. Introduction

Mucopolysaccharidosis type IVa (MPS IVa, Morquio–Brailsford syndrome; OMIM 253000) is a rare inherited autosomal recessive lysosomal storage disorder caused by a deficiency of N-acetylgalactosamine-6-sulfate sulfatase (GALNS). This results in impaired degradation of the glycosaminoglycans (GAGs) keratan sulfate (KS) and chondroitin-6-sulfate (C6S). Consequently, these undegraded GAGs accumulate in the extracellular matrix of tissues, leading to the characteristic systemic skeletal dysplasia and connective tissue abnormalities [1,2].

The enzyme GALNS is encoded by the GALNS gene, located on chromosome 16q24.3 [3,4]. To date, up to 450 pathogenic variants have been identified [5]. Most of these are private variants, and for the majority of them, no clear genotype–phenotype correlation has been established.

There is a broad clinical spectrum of MPS IVa phenotypes, ranging from classic early-onset to attenuated late-onset forms. Patients with MPS IVa usually appear normal at birth, with more than 70% presenting within the first three years of life with signs of skeletal dysplasia. The first symptoms mainly include growth arrest resulting in dwarfism, thoracic deformities such as pectus carinatum and kyphoscoliosis, as well as genu valgum and coxa valga. Further typical clinical manifestations include odontoid hypoplasia, flaring of the ribs, stiff and hypermobile joints, and an abnormal gait. Non-skeletal manifestations include hearing impairment, visual impairment, spinal cord compression, and respiratory and cardiac disease [6,7,8].

Patients with severe forms often do not survive beyond the second to third decade of life, primarily because of respiratory insufficiency [9]. Patients with milder manifestations of MPS IVa have been reported to survive into the seventh decade of life [10].

Cardiovascular problems reported in MPS IVa patients include heart valve disease, left ventricular hypertrophy, aortic root dilatation, coronary artery sclerosis, and increased heart rates [11,12,13]. Although vascular shape alterations, particularly involving the vertebrobasilar and brachiocephalic arteries, have been reported in MPS IVa [14,15], previous studies have focused mainly on selected vascular territories or on the relationship between vascular anatomy and airway narrowing. Quantitative three-dimensional assessment of the full aorta and extracranial carotid arteries with comparison to age- and sex-matched controls remains limited.

We therefore aimed to quantify aortic and carotid tortuosity and kinking using 3D magnetic resonance (MR) angiography and to compare tortuosity indices with an age- and sex-matched external imaging control group. Given the small number of eligible patients, the analyses were considered exploratory.

## 2. Materials and Methods

### 2.1. Patients

We retrospectively analyzed six patients with enzymatically and/or genetically confirmed MPS IVa who were followed at the outpatient clinic of Villa Metabolica, the Metabolic Unit of the University Medical Center Mainz, and underwent clinically indicated MR angiography of the aorta and supraaortic vessels in 2015. Two of the six subjects were excluded from the analyses because of motion artefacts, leaving four evaluable patients. As a result of this highly selected cohort, the applicability of the findings to the broader patient population is inherently limited. All patients and/or their legal guardians provided written informed consent for data analysis. Because no MR angiographic examinations of healthy subjects were available in our records, age- and sex-matched polytrauma patients without documented vascular injuries or abnormalities were used as external imaging controls. The patients with MPS IVa underwent MR angiography, whereas controls underwent CT angiography; therefore, between-group comparisons are exploratory. According to local regulations, approval of the local independent ethics committee was not necessary due to the retrospective study design.

### 2.2. MR Protocol

All patients and/or their legal guardians provided written informed consent for the clinically indicated MR examinations, which were performed on a 1.5T scanner (Magnetom Avanto^®^, Siemens Healthineers, Erlangen, Germany). Contrast-enhanced MR angiography of the entire aorta, including the supraaortic vessels up to the skull base, was performed using a standard 3D spoiled gradient-echo sequence (repetition time: 3.21 ms; echo time: 1.15 ms; flip angle: 25°; field of view: 500 mm; slice thickness: 1.2 mm; matrix: 512 × 512). Integrated spine coil elements, an additional neck coil, and a standard anterior phased-array body coil were used for signal detection. A test bolus of 1 mL gadoteric acid (0.5 mmol/ml, Dotarem^®^, Guerbet, Villepinte, France) was administered to determine the individual delay until contrast enhancement of the ascending aorta. For MR angiography, a weight-adjusted dose of gadoteric acid (0.2 mL/kg body weight) was administered via an automated injection pump at a flow rate of 2–3 mL/s, depending on the size of the inserted peripheral venous catheter.

### 2.3. Evaluation of the MR Angiographies

**Figure 1:**
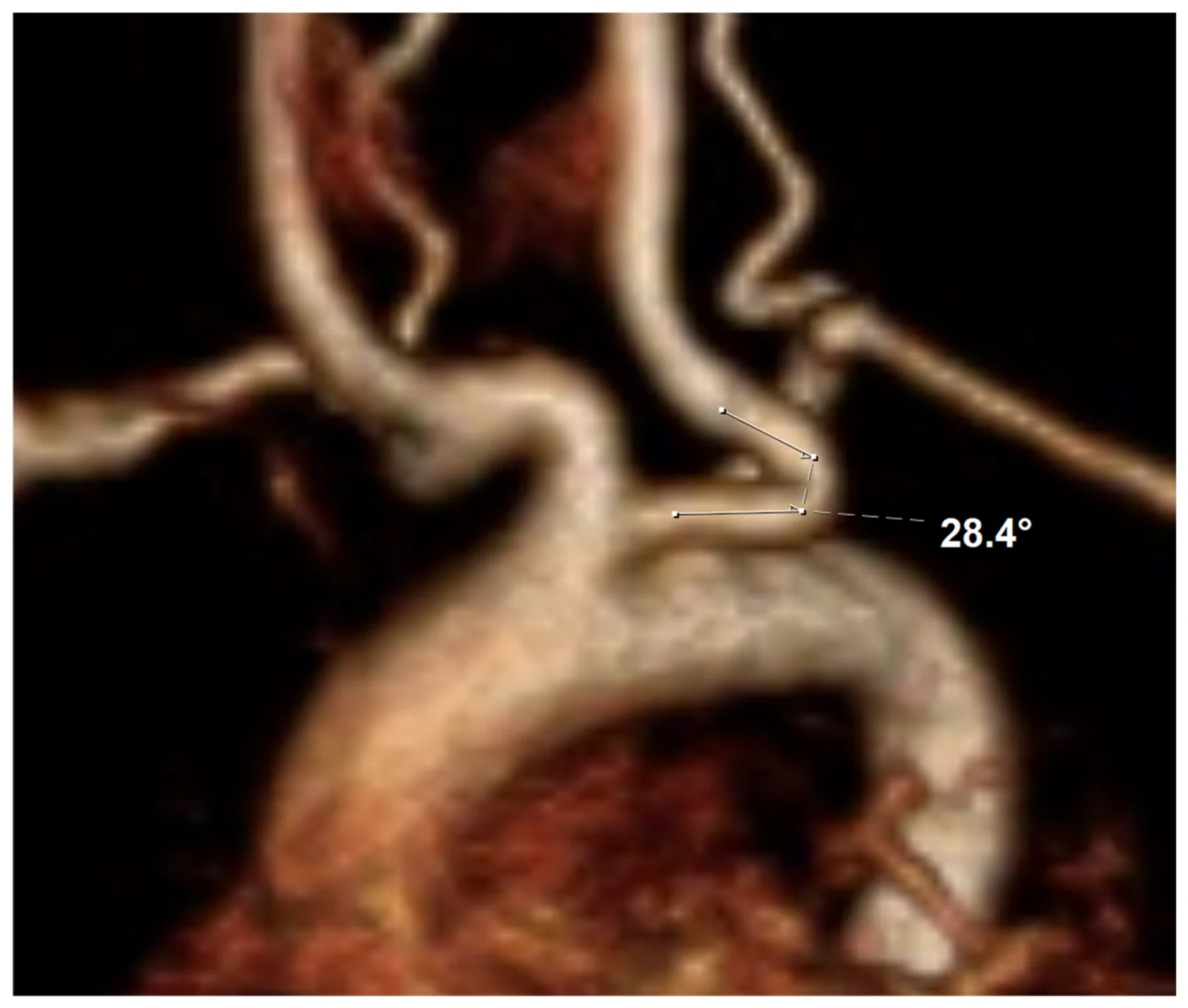
Enlarged part of an anterior 3D reconstruction derived from MR angiography that demonstrates a kinking site of the left common carotid artery, which originates from a common carotid trunk (patient 1). The kinking angle is measured between the two adjacent vessel segments.

All MR angiographies were assessed by two of the authors (A.L., a pediatric radiologist with more than 15 years of experience in pediatric MR reading, and F. S., a 5th-year radiology resident) independently. We evaluated the aorta (from the annulus to the iliac bifurcation), as well as the common and internal carotid arteries for kinking and tortuosity. Distinct kinking sites were identified morphologically as non-anatomical angulations by the radiologist and counted individually. For each vessel, the most pronounced kinking angle was measured; for vessels with multiple kinkings, the minimum angle is reported. Smaller angles indicate more severe kinking, with 0° indicating a complete U-turn of the vessel. According to the classification proposed by Saba et al. [16], carotid artery kinking was graded as mild for angles ≥60°, moderate for angles ≥30° and < 60°, and severe for angles <30°. A sample measurement is shown in Figure 1.

For the aorta, a study-specific adapted threshold based on Belvroy et al. [17] was used to achieve better comparability with the aforementioned carotid artery classification, with angles <120° (the reciprocal value of the original definition) defined as a non-physiological kinking. In addition, the minimum cross-sectional area (CSA) of the descending aorta at a kinking site was measured perpendicular to the vessel centerline and related to the maximum CSA in an adjacent aortic segment to assess anatomical narrowing. Due to limited spatial resolution, CSA measurements were not performed in the carotid arteries.

**Figure 2:**
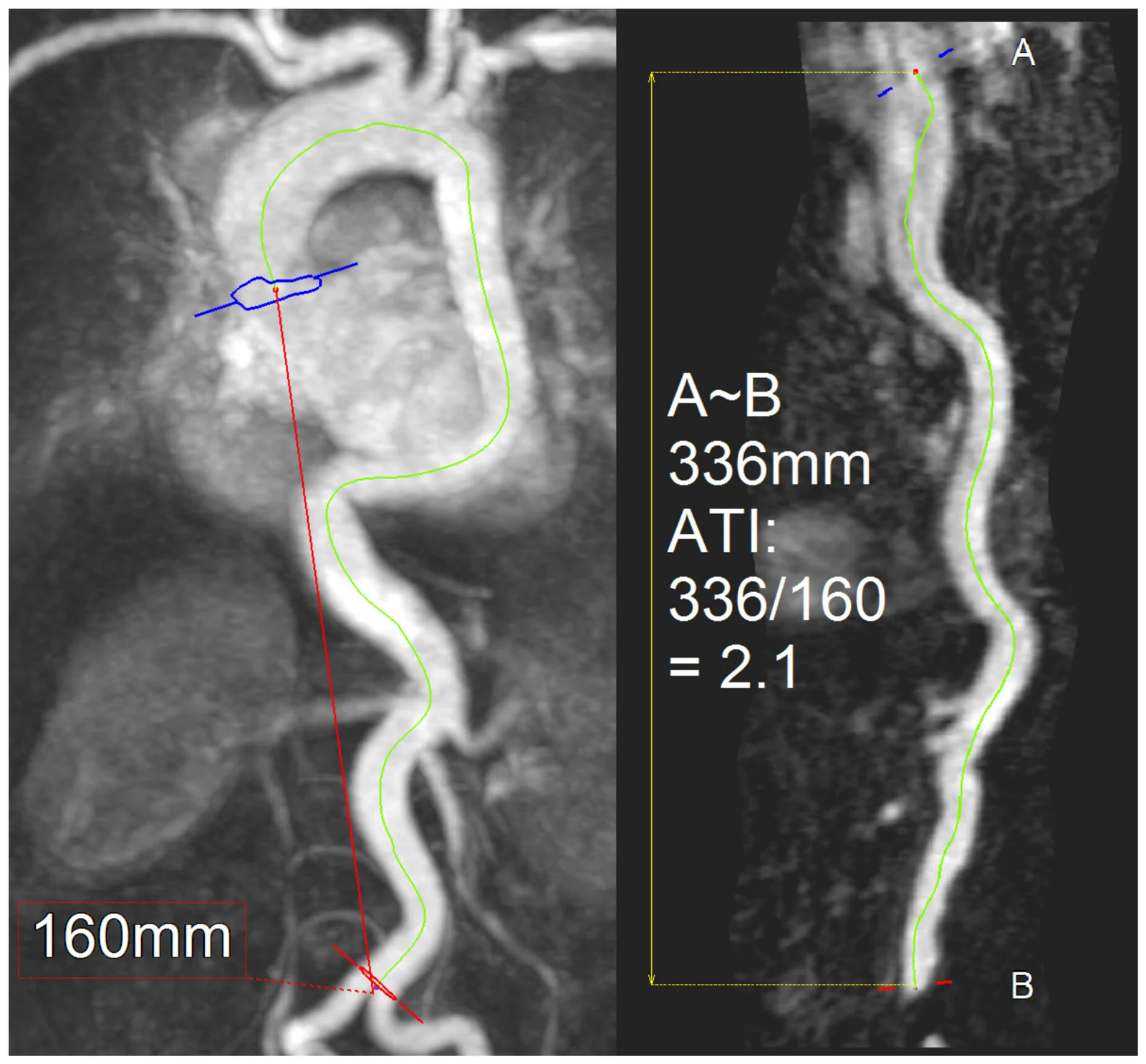
Anterior maximum-intensity projection and curved multiplanar reconstruction of the aorta. The aortic tortuosity index (ATI) is calculated by dividing the length of the curved centerline (336 mm) by the geometric length from the annulus (A) to the aortic bifurcation (B, 160 mm); these data result in an ATI of 2.1.

**Figure 3:**
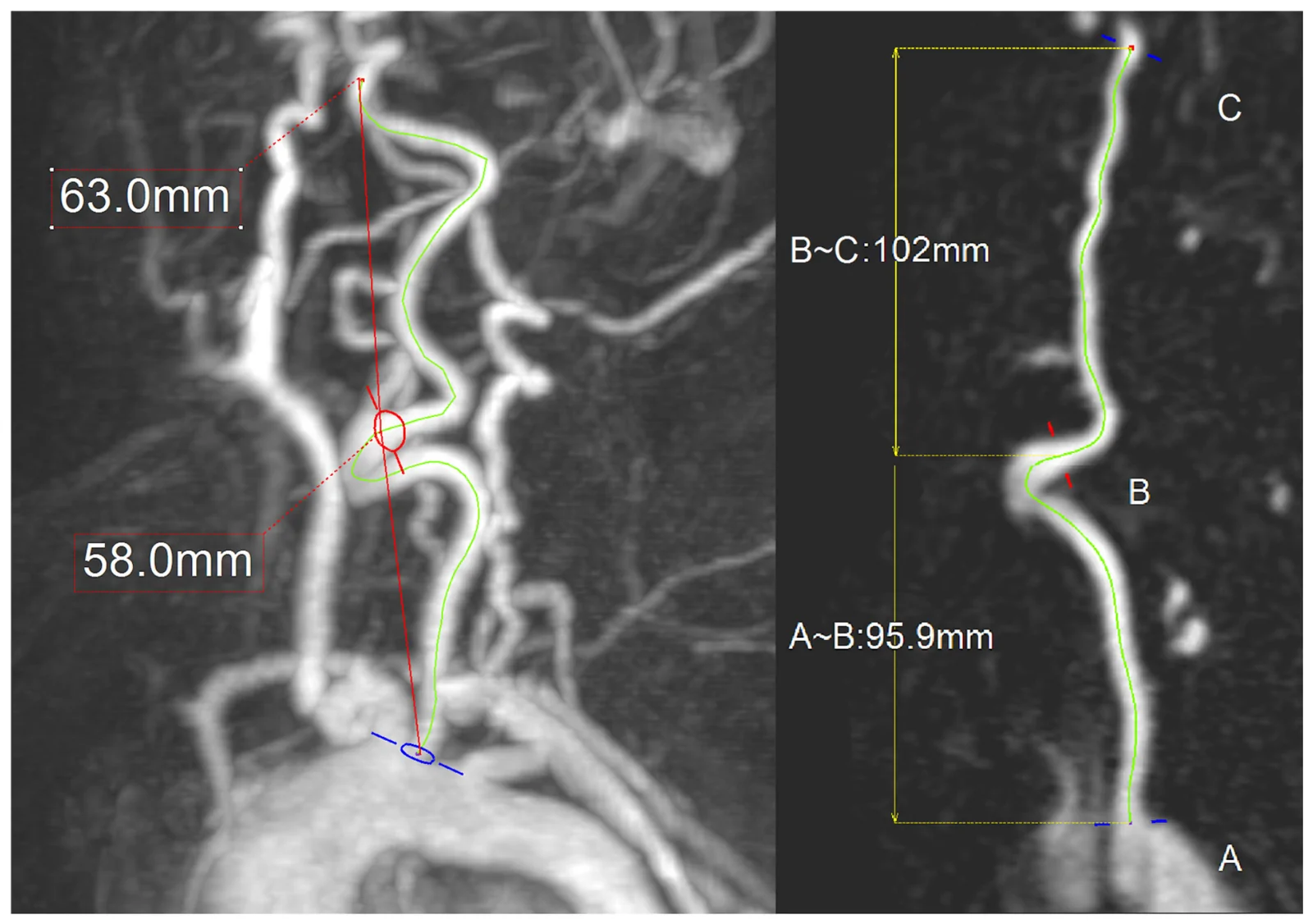
Left-anterior-oblique maximum-intensity projection and curved multiplanar reconstruction of the left carotid artery. The tortuosity index (TI) is calculated by dividing the length of the curved centerlines (96 and 102 mm) by the geometric lengths from the carotid trunk (A) to the bifurcation (B, 58 mm), and from the bifurcation to the skull base (C, 63 mm); these data result in a TI of 1.66 for the common, and 1.62 for the internal carotid artery.

For the aorta, the aortic tortuosity index proposed by Franken et al. [18] was calculated. For the carotid arteries, a tortuosity index was calculated as described by Choudry et al. [19]. Both indices measure the relationship between the length of the respective vessel’s curved centerline and its geometric length. All measurements were performed using Aquarius iNtuition^®^ (version 4.4.12, TeraRecon Inc., Foster City, CA, USA) or syngo.via^®^ (version 11.4, Siemens Healthineers, Erlangen, Germany) software. Sample measurements of the tortuosity indices of the aorta and carotid arteries are shown in Figure 2 and Figure 3.

### 2.4. Retrospective Evaluation of the Polytrauma CT Scans

A total of 12 polytrauma CT scans (three age- and gender-matched control patients per subject with MPS IVa) were retrospectively evaluated independently by the two aforementioned authors (A. L. and F. S.). These scans were performed on a 256-slice CT scanner (Brilliance iCT^®^, Philips Healthcare, Best, The Netherlands) between 2015 and 2019. We narrowed the time frame as much as possible to the period in which the MR angiographies were performed. However, due to the limited number of adolescent polytrauma patients who underwent CT, we could not restrict the analysis to cases from 2015 alone. We used a standardized imaging protocol covering the whole torso and neck (up to the skull base) using a split-bolus technique with a standard dose of 120 mL (or 1.5 mL per estimated kg body weight in adolescent patients) iodinated contrast agent (Imeron 400^®^, Bracco Imaging S.p.A., Milan, Italy). Measurements of aortic and carotid tortuosity were performed in analogy to the evaluation of the MR angiographies as described in Section 2.3.

### 2.5. Statistics

All computations were performed using SPSS (version 29.0, IBM, Armonk, NY, USA). Descriptive statistics were calculated to characterize all patients with MPS IVa as well as the corresponding control patients. The comparison of tortuosity indices between patients and controls was tested for significance using the Mann–Whitney U-test. Interobserver agreement for the tortuosity indices was evaluated using the intra-class correlation coefficient (ICC, two-way mixed model, absolute agreement, single measures) [20]. ICCs > 0.75 or >0.9 indicated a good and excellent agreement, respectively. A *p*-value lower than 0.05 was considered significant in all analyses, which were exploratory; no adjustments for multiple testing were made.

## 3. Results

### 3.1. Patient Characteristics

The four evaluable patients were male, aged 17.63 ± 4.44 years (range: 12.7–22.4 years) at the time of MR angiography, and all had a severe MPS IVa phenotype. Their heights ranged from 96 to 103 cm. Cardiovascular findings included arterial hypertension with sinus tachycardia in one patient, aortic valve stenosis in one, and aortic insufficiency in one; the fourth patient had no reported cardiovascular symptoms. Three patients had restrictive lung disease, and one of these also had obstructive sleep apnea. Individual clinical characteristics, GALNS variants, and enzyme activity are summarized in Table 1; additional clinical details are provided in Supplementary Information S1.

### 3.2. Evaluation of Imaging Data

Aberrant vessel courses were detected in all patients with MPS IVa. Two patients had a bicarotid trunk, one patient had an ectatic left carotid bulb, and one patient showed no anomalies of the aortic arch or supraaortic trunks. All patients presented with multiple kinkings of the descending aorta, with a maximum of five kinkings per patient. These kinkings did not lead to relevant aortic stenosis. All patients also exhibited carotid artery kinkings, with the most severe kinking angles observed in the internal carotid arteries. These results are summarized in Table 2.

**Figure 4:**
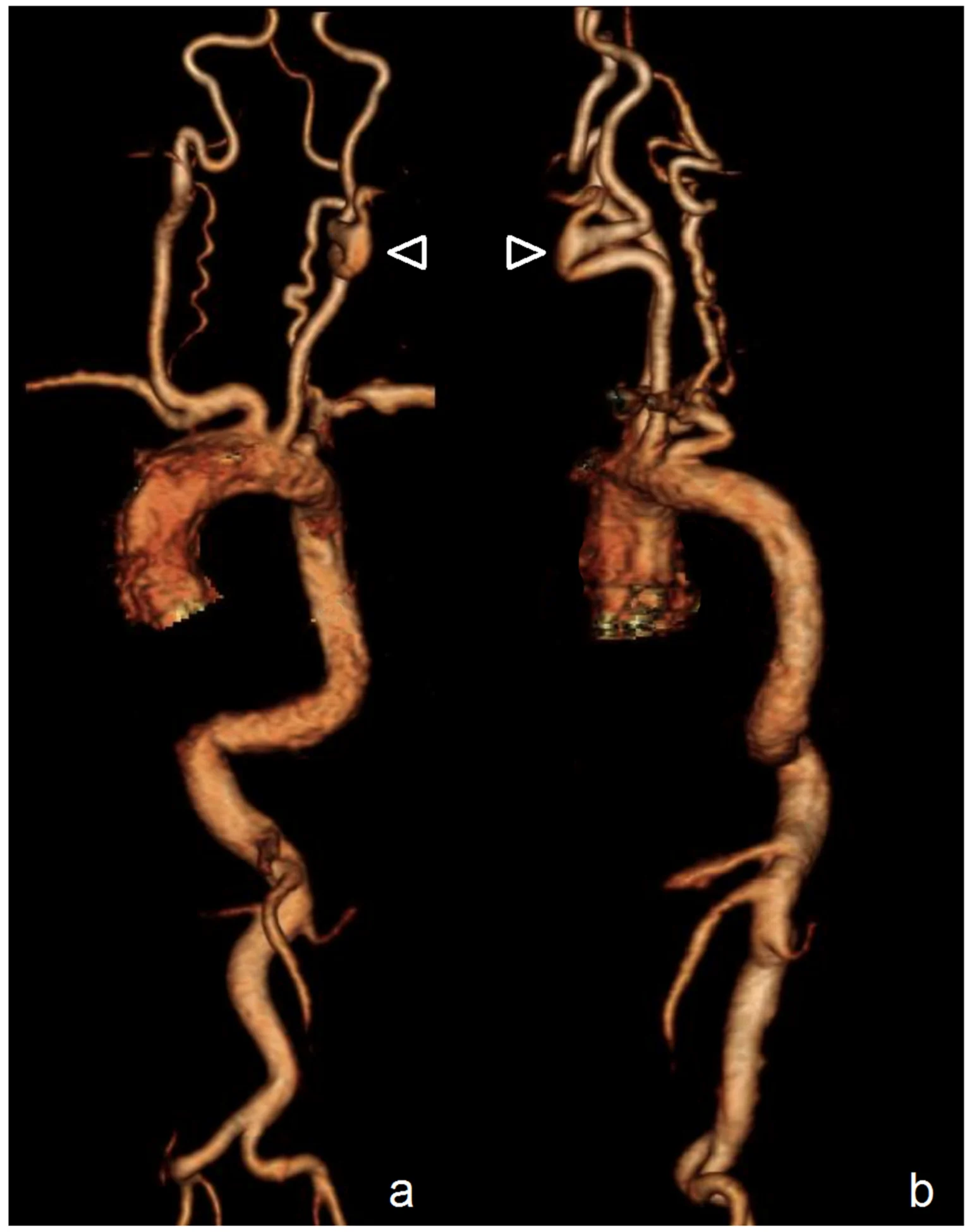
3D reconstruction of the aorta and supraaortic vessels derived from MR angiography (patient 4). An anterior (panel (**a**)) and left-lateral view (panel (**b**)) is shown. Multiple kinkings of the descending aorta are depicted, as well as a severe kinking involving the distal left common carotid artery, the carotid bifurcation, and the proximal internal carotid artery in the anterior direction (arrowheads).

Aortic tortuosity ranged from 2.01 to 2.68 (Table 2), exceeding the published mean ± standard deviation range of 1.72–1.92 reported by Franken et al. [18]. Tortuosity indices were also descriptively higher than published reference values in both common and internal carotid arteries. Tortuosity indices of the right common carotid artery ranged from 1.22 to 1.47 (Table 2; reference range adapted from Choudhry et al. [19] [mean ± standard deviation]: 1.0–1.2), whereas those of the left common carotid artery ranged from 1.32 to 1.66 (reference range: 1.01–1.21). Tortuosity indices of the right internal carotid artery ranged from 1.62 to 2.12 (reference range: 1.1–1.3), whereas those of the left internal carotid artery ranged from 1.61 to 1.71 (reference range: 1.09–1.29). The tortuosity indices did not consistently differ between patients with a minimum residual GALNS activity and those without any enzyme activity (mean TI aorta: 2.42 ± 0.37 versus 2.05 ± 0.06 right common carotid: 1.35 ± 0.18 versus 1.44 ± 0.21; right internal carotid: 1.87 ± 0.35 versus 1.79 ± 0.19; left common carotid: 1.37 ± 0.07 versus 1.51 ± 0.22; left internal carotid: 1.63 ± 0.21 versus 1.67 ± 0.06). However, the cohort was too small to assess genotype- or enzyme-activity associations meaningfully. An example of aberrant vessel courses (patient 4) is shown in Figure 4.

### 3.3. Comparison of Patients with MPS IVa Versus Controls and Interobserver Agreement

We included 12 male control patients aged 17.8 ± 4.17 years (range: 12 to 22 years, *p* versus patients with MPS IVa = 0.77) in our analysis. Tortuosity indices as assessed by the two observers are presented in Table 3.

Interobserver agreement for the tortuosity indices ranged from good to excellent, with intraclass correlation coefficients (ICCs) of 0.954 (95% confidence interval [CI]: 0.871–0.984, *p* < 0.001) for the aorta, of 0.969 (95% CI: 0.916–0.989, *p* < 0.001) for the right common carotid artery, of 0.905 (95% CI: 0.697–0.968, *p* < 0.001) for the right internal carotid artery, of 0.886 (95%-CI: 0.699–0.959, *p* < 0.001) for the left common carotid artery, and of 0.882 (95% CI: 0.626–0.96, *p* < 0.001) for the left internal carotid artery.

## 4. Discussion

This study was designed to elucidate changes in the aorta and carotid arteries using 3D MR angiography in patients with MPS IVa. Various cardiovascular abnormalities have been reported in different MPS animal models [21,22,23]. Increased aortic wall thickness, aortic stiffness, aortic coarctation, aortic root dilatation, aneurysms, and coronary artery disease have been described in patients with different MPS types [24,25,26,27]. Histological examinations also revealed increased carotid intima-media thickness in these patients [24,26].

In this exploratory comparison, tortuosity indices were higher in the MPS IVa cohort than in the age- and sex-matched imaging controls, and multiple kinking sites were observed in the aorta and carotid arteries. Given that only four patients were evaluable and the patient and control groups were imaged with different modalities, these group differences should be interpreted cautiously. Of note, the TI values in our controls were lower than those reported by Franken et al. [18] and Choudhry et al. [19], which might be attributable in part to the younger age range of our cohort. We observed up to five aortic kinking sites per patient, as well as kinking of the carotid arteries, with the most pronounced carotid angulations in the internal carotid arteries. Tanyildizi et al. [14] demonstrated significantly increased tortuosity and elongation of the vertebrobasilar arteries in the majority of patients with MPS IVa, while Averill et al. [28] reported tortuosity of the brachiocephalic artery in 95% of patients, as well as frequent tortuosity of the aortic arch (78%) and descending thoracic aorta (84%). This is in line with the findings of Tomatsu et al. [15], who reported that tracheal narrowing is a frequent sequela of compression from the crossing tortuous brachiocephalic artery. Taken together with the aforementioned previous reports of vertebrobasilar and brachiocephalic vascular abnormalities, our findings raise the possibility that increased tortuosity may represent a broader vascular manifestation of MPS IVa, but the prevalence and clinical significance cannot be established by the present cohort.

Anatomical narrowing of the descending aorta was present at the kinking sites with minimum-to-maximum CSA ratios ranging from 0.60 to 0.89, corresponding to an estimated area reduction of approximately 11–40%. Published physiological and model-based studies suggest that larger area reductions (>60%) may be associated with more substantial flow impairment [29,30]. These findings cannot be directly extrapolated to MPS IVa, and hemodynamic consequences were not directly assessed in this study. The observed CSA ratios therefore describe anatomical narrowing only and should not be interpreted as evidence for or against clinically relevant flow obstruction.

No consistent descriptive pattern relating residual GALNS enzyme activity to tortuosity indices was apparent: the ATI and TI of the right common carotid artery were numerically higher in patients with little residual activity than in those without any residual activity, whereas the TIs of the right internal and left common and internal carotid arteries were numerically higher in the latter group. However, the cohort was too small to permit meaningful assessment of genotype- or enzyme-activity associations. Furthermore, an in vitro expression study reported an association between residual GALNS activity below 1% of wild-type activity and severe MPS IVA phenotypes [31], and all four patients included in our study had an enzyme activity below this threshold. In addition, the evaluation of residual enzyme activity as a marker of the biochemical phenotype is limited by two factors: first, enzyme activity is measured in vitro, and second, an artificial substrate (4-methylumbelliferyl) is used for this measurement.

The exact cause of arterial tortuosity and kinking in patients with MPS IVa remains incompletely understood. In general, arterial tortuosity has been associated with a loss of elasticity due to aging, hypertension, and atherosclerosis [32], but may also occur in genetic arteriopathies [33]. Vascular changes in MPS IVa have been attributed to an imbalanced growth of the aorta in relation to the shortened thorax, and to GAG deposition within the vascular walls [28]. Still, histopathological or molecular markers for this hypothesis have not been assessed in the aforementioned study. However, postmortem histopathological examination of an adolescent patient with MPS IVa revealed thickening of the aortic intima and media, accompanied by macrophage infiltration, C6S storage, and disruption of elastin fibrils [34]. Elastin dysfunction and alterations in proteoglycans have been proposed to affect arterial function in patients with MPS IVa. This may result in increased carotid elasticity, in contrast to other MPS types, which are characterized by reduced carotid elasticity [35]. Recent work has also investigated extracellular-matrix biomarkers associated with cardiovascular disease in MPS IVa [36], although these findings do not directly establish a mechanism for the tortuosity observed here.

In an MPS IVa rat model, untreated animals exhibited lysosomal distension of smooth-muscle fibers in the aortic wall, accompanied by electrolucent vacuoles in aortic fibers on ultrastructural examination [37]. Furthermore, immune-modulated inflammatory processes secondary to storage phenomena have been shown to contribute to aortic changes in MPS I animal models [21,38]; however, it remains unclear whether these mechanisms can be applied to patients with MPS IVa. Collectively, these factors may contribute to the development of kinking and tortuosity of the aorta and other arteries in patients with MPS IVa.

Imaging of vascular abnormalities remains challenging, as these patients frequently present with respiratory distress in the supine position due to upper-airway obstruction, tracheal narrowing, restrictive pulmonary disease, and cervical spine instability, particularly when sedation is used [39]. As a result of these conditions, two of the six patients in our study were unable to tolerate the MR examination, which offers the general advantage of being radiation-free but has the drawback of longer examination times without motion. Because of its faster acquisition and despite the use of ionizing radiation, CT angiography may be an alternative for patients who are unable to remain motionless for extended periods. Another reason to perform CT instead of MRI would be the simultaneous evaluation of the tracheobronchial system, if a stenosis is suspected.

Still, in the four patients with successful MR imaging data available for evaluation, aortic and carotid artery tortuosity indices could be assessed with a good-to-excellent interobserver agreement, a finding that—to our knowledge—has not been previously assessed in the literature for patients with MPS IVa. Compared with the study by Franken et al. [18], which included patients with Marfan syndrome, we demonstrated a similar interobserver agreement for the ATI (ICC 0.955 versus 0.954 in our study). However, while these ICCs support measurement reproducibility under the study protocol, they do not establish clinical validity or prognostic utility.

The current guidelines recommend obtaining electrocardiograms and echocardiograms every 3 years for cardiac screening [6,40]. However, specific recommendations for the systematic assessment of arterial abnormalities, such as aortic or carotid artery morphology, are currently lacking. No validated ATI thresholds linking specific ranges of tortuosity to cardiovascular risk are currently established for MPS IVa; therefore, we did not stratify patients according to arbitrary ATI categories. The clinical implications of carotid tortuosity in MPS IVa also remain unclear. Our study demonstrated increased carotid artery tortuosity in patients with MPS IVa consistent with a previously published case report, in which widespread vasculopathy is described [41]. However, the potential effects of carotid tortuosity on cerebral perfusion and cerebrovascular risk have not been systematically assessed in this disease. In the general population, carotid tortuosity and kinking have been identified as potential contributors to altered cerebral hemodynamics [42] and cerebrovascular disease [43]. However, a recent systematic review found that the association between specific cervical artery tortuosity patterns and dissection remains inconclusive [44]. In addition, these findings cannot be directly extrapolated to MPS IVa.

Our study has several limitations. First, only four patients were evaluable; all were male and had a severe phenotype. The analyses are therefore exploratory and cannot establish population prevalence, disease-specific causation, or prognostic thresholds. Second, although we described vascular variations in terms of kinking and tortuosity, their impact on the clinical course remains unclear, as we did not assess whether these findings influenced clinical decision-making. Long-term follow-up studies are needed to evaluate the progression of vascular changes over time, with particular attention to the potential development of complications such as aneurysms or relevant stenoses. The MR angiographic examinations were performed in 2015 and were retrospectively analyzed several years later. Thus, the findings reflect vascular morphology at a single historical time point, and no serial imaging was available to assess progression over time. Third, the four MPS IVa patients had marked short stature (96–103 cm) and severe skeletal dysplasia. Thoracic shortening and altered skeletal anatomy may influence vessel course and geometric reference distances, and we cannot distinguish intrinsic vascular-wall abnormalities from secondary geometric effects of skeletal deformity. The height of the polytrauma control patients was not available retrospectively, so body size could not be matched or adjusted for. Furthermore, MPS IVa patients were examined with MR angiography whereas controls underwent CT angiography. Differences in spatial resolution, vessel depiction, and image reconstruction may affect centerline extraction and TI calculations. No paired MR/CT examinations were available, so the comparability of the two modalities for our specific whole-aorta and carotid TI measurements could not be directly validated. Finally, hemodynamic effects were not directly assessed. A recent systematic review similarly concluded that the role of imaging-based vascular risk assessment in MPS remains underdefined [27].

## 5. Conclusions

In this exploratory comparative imaging study, the four evaluable patients with MPS IVa showed pronounced tortuosity and kinking of the aorta and carotid arteries compared with age- and sex-matched controls. These findings extend previous observations of vascular shape abnormalities in MPS IVa [14,28], and generate the hypothesis that altered large-vessel geometry may be part of its broader vascular phenotype. However, the small sample size, the use of different imaging modalities in patients and controls, and potential effects of severe skeletal dysplasia limit causal interpretation. The prevalence and clinical or hemodynamic significance of these findings remain uncertain, and our data do not establish indications or intervals for dedicated vascular imaging. Larger, preferably multicenter longitudinal studies are needed to determine the prevalence, progression, and clinical relevance of vascular tortuosity in MPS IVa and to identify patients who may benefit from dedicated vascular imaging. Contrast-enhanced 3D MR angiography provides a radiation-free means of characterizing vascular morphology, which is preferable in patients who can undergo the examination without relevant motion or the need for sedation.

## Figures and Tables

**Table 1:** Clinical data at the time of the MR examination.

	Patient 1	Patient 2	Patient 3	Patient 4
age [years]	12.7	22.4	15.3	20.1
height [cm]	96	100	99	103
consanguinity	yes	yes	no	yes
age at treatment initiation [years]	12.1	19.5	14.7	17.4
phenotype	severe	severe	severe	severe
cardiovascular symptoms	none	AHT ST	AVS	AI
pulmonary symptoms	none	RLD OSAS	RLD	RLD
lung function	FVC: 88% FEV1: 102%	FVC: 65% FEV1: 41%	FVC: 51% FEV1: 60%	FVC: 40% FEV1: 30%
GALNS variant allele 1 (NM_000512.4)	c.867C>G p.(Asn289Lys)	not available	c.860C>T p.(Ser287Leu)	c.1168delC p.(Leu390Ter)
GALNS variant allele 2 (NM_000512.4)	c.867C>G p.(Asn289Lys)	not available	c.1168delC p.(Leu390Ter)	c.1168delC p.(Leu390Ter)
enzymatic activity of GALNS	0.07%	0.04%	0%	0%

AHT, arterial hypertension; AI, aortic insufficiency; AVS, aortic valve stenosis; OSAS, obstructive sleep apnea syndrome; RLD, restrictive lung disease; ST, sinus tachycardia.

**Table 2:** Summary of measures derived from MR angiography (data from observer 1).

	Patient 1	Patient 2	Patient 3	Patient 4
aorta				
- number of kinkings	2	5	2	5
- min. kinking angle [°]	90	50	83	50
- minimum CSA [mm^2^]	83	236	93	121
- maximum CSA [mm^2^]	139	264	133	200
- ratio min./max. CSA	0.6	0.89	0.7	0.61
- tortuosity index	2.16	2.68	2.01	2.1
right common carotid				
- number of kinkings	1	1	1	1
- min. kinking angle [°]	52	32	63	74
- tortuosity index	1.22	1.47	1.42	1.45
right internal carotid				
- number of kinkings	2	2	3	3
- min. kinking angle [°]	0	18	5	3
- tortuosity index	1.62	2.12	1.65	1.92
left common carotid				
- number of kinkings	1	1	0	1
- min. kinking angle [°]	28	86	n/a	67
- tortuosity index	1.32	1.42	1.35	1.66
left internal carotid				
- number of kinkings	3	2	3	2
- min. kinking angle [°]	25	24	6	8
- tortuosity index	1.64	1.61	1.71	1.62

CSA: cross-sectional area.

**Table 3:** Comparison of tortuosity indices (mean ± standard deviation) between patients and controls as assessed by the two observers.

	Observer 1			Observer 2		
	Patients	Controls	*p* Value	Patients	Controls	*p* Value
aorta	2.24 ± 0.3	1.65 ± 0.07	0.001	2.22 ± 0.27	1.62 ± 0.1	0.001
right common carotid	1.39 ± 0.12	1.06 ± 0.05	0.001	1.36 ± 0.1	1.06 ± 0.04	0.001
right internal carotid	1.83 ± 0.24	1.13 ± 0.11	0.001	1.66 ± 0.21	1.09 ± 0.05	0.001
left common carotid	1.44 ± 0.15	1.05 ± 0.04	0.001	1.33 ± 0.07	1.04 ± 0.03	0.001
left internal carotid	1.65 ± 0.05	1.13 ± 0.07	0.001	1.51 ± 0.14	1.1 ± 0.06	0.001

## Data Availability

A.L., G.S. and J.B.H. had full access to all the data in the study and take responsibility for the integrity of the data and the accuracy of the data analysis. The data presented in this study are available on request from the corresponding authors. The data are not publicly available due to privacy (medical data).

## Supplementary Materials

The following supporting information can be downloaded at https://www.mdpi.com/article/10.3390/diagnostics16213627/s1. Supplementary Information S1—Additional clinical details of the study cohort [45].

## Abbreviations

The following abbreviations are used in this manuscript:

AHT	arterial hypertension
AI	aortic insufficiency
(A)TI	(aortic) tortuosity index
AVS	aortic valve stenosis
C6S	chondroitin-6-sulfate
CI	confidence interval
CSA	cross-sectional area
CT	computed tomography
GAG	glycosaminoglycan
GALNS	N-acetylgalactosamine-6-sulfate sulfatase
ICC	intraclass correlation coefficient
KS	keratan sulfate
MPS	mucopolysaccharidosis
MR	magnetic resonance
OSAS	obstructive sleep apnea syndrome
RLD	restrictive lung disease
ST	sinus tachycardia
