# Supplementary material for: Quantitative 3-Dimensional Characterization of Aortic and Carotid Artery Tortuosity in Mucopolysaccharidosis Type IVa: An Exploratory Comparative Imaging Study
Source: Diagnostics (Basel). 2026 Nov 3;16(21):3627. doi: 10.3390/diagnostics16213627 (PMC13762197; doi:10.3390/diagnostics16213627)
Supplement: Supplementary file 1 [file diagnostics-16-03627-s001.zip › Supplementary Information S1.pdf]

Supplementary Information S1 – Detailed Case Reports

Patient 1

He is the first child of consanguineous parents of Turkish origin. Two younger siblings are not affected by MPS IVa.

The patient was born preterm by spontaneous delivery at 35 weeks of gestation. The postnatal period was uneventful. The first clinical symptoms manifested at the age of 1 year with thoracic deformities, followed by growth retardation as well as gait abnormalities due to genua valga and hip dysplasia. A delay in speech development caused by hearing impairment was diagnosed at the age of 5.5 years; hearing aids were placed at the age of 6 years. Surgical interventions included insertion of T-tubes at the age of 2.5 years, adenotomy and simultaneous craniospinal decompression at the age of 4.7 years.

MPS IVa was diagnosed at the age of 3.8 years. GALNS activity measured in leukocytes was decreased to 0.07% residual activity. Mutation analysis of the GALNS gene revealed homozygosity for the novel missense mutation c.867C>G (exon 8; NM_000512.4:c.[867C>G];[867C>G], p.[(Asn289Lys)];[(Asn289Lys)]).

Enzyme replacement therapy with elosulfase alfa was started at the age of 12.1 years.

Patient 2

He is the fourth child of consanguineous parents of Turkish origin. One older sister was affected by MPS IVa and died at the age of 4 years due to acute craniospinal compression. Two other female siblings are healthy.

Pregnancy was complicated by acute gestosis. The boy was born at 36 weeks of gestation, and he presented with congenital clubfeet. Due to the positive family history, urinary GAG screening was performed at the age of 4 weeks, which yielded a negative result. At the age of 3 months, the boy suffered from urosepsis due to vesicoureteral reflux. At the age of 15 months, he presented with gait abnormalities and thoracic deformities. Growth retardation started during the second year of life. Hearing impairment requiring hearing aids was diagnosed at the age of 13 years. Surgical interventions included tenotomy of both Achilles tendons at the age of 0.4 years and craniospinal decompression at the ages of 5.4 and 14 years.

MPS IVa was diagnosed at the age of 1.75 years. Enzymatic GALNS activity measured in leukocytes was decreased to 0.04% residual activity. GALNS gene analysis was not performed, as it was refused by the patient.

Enzyme replacement therapy with elosulfase alfa was started at the age of 19.5 years.

Patient 3

He is a male from a twin pregnancy of non-consanguineous parents of Turkish origin. The other twin and two younger siblings are healthy.

The patient was born preterm at 32 weeks of gestation. The postnatal period was complicated by prematurity and respiratory problems.

At the age of 0.5 years, the parents noticed lumbar kyphosis. During the second year of life, the patient developed growth retardation and genua valga. Hearing impairment was diagnosed at the age of 10 years, resulting in the use of hearing aids at the age of 11.7 years. Surgical interventions included T-tube insertion at the ages of 7.1 and 9.7 years, as well as craniospinal decompressions at the ages of 2.8, 6.6, and 12.7 years.

MPS IVa was diagnosed at the age of 3 years. GALNS activity measured in leukocytes showed no residual activity. Mutation analysis revealed compound heterozygosity for c.860C>T (exon 8) and c.1168delC (exon 11; (NM_000512.4:c.860C>T(;)1168delC, p.(Ser287Leu)(;)(Leu390Ter)), both previously described muations.

Enzyme replacement therapy with elosulfase alfa was started at the age of 14.7 years.

Patient 4

He is the third child of consanguineous parents of Turkish origin. One older sister was affected by MPS IVa and died at the age of 21 years due to acute respiratory failure; one older brother is healthy.

Pregnancy was uneventful. The boy was born at 40 weeks of gestation. The postnatal period was uneventful.

At the age of 2 years, the patient presented with joint and thoracic deformities as well as growth retardation. Due to hearing impairment, hearing aids had to be fitted. Surgical interventions included insertion of T-tubes at the age of 16 years.

MPS IVa was diagnosed at the age of 2.75 years. Enzymatic GALNS analysis measured in leukocytes showed no residual activity. Mutation analysis revealed homozygosity for c.1168delC (exon 11; (NM_000512.4: c.[1168delC];[1168delC], p.[(Leu390Ter)];[(Leu390Ter)]), the same deletion as identified in patient 3.

Enzyme replacement therapy with elosulfase alfa was started at the age of 17.4 years.
